# Prevalence and risk factors of obstructive sleep apnea in depression: an observational retrospective study

**DOI:** 10.3389/fpsyt.2025.1568830

**Published:** 2025-04-07

**Authors:** Xiujuan Chen, Zhengfa Qiu, Changzhou Hu, Zhiwang Liu

**Affiliations:** Department of Psychiatry, Affiliated Kangning Hospital of Ningbo University (Ningbo Kangning Hospital), Ningbo, Zhejiang, China

**Keywords:** depression, sleep apnea, obstructive, risk factors, polysomnography, retrospective study

## Abstract

**Background:**

A substantial number of previous studies have concentrated on the prevalence of depression among patients with obstructive sleep apnea (OSA). However, research regarding the prevalence of OSA among patients with depression remains relatively scarce. The aim of this study was to determine the prevalence of OSA among patients with depression and to identify the associated risk factors.

**Method:**

A single-center retrospective chart review was conducted. The research focused on patients diagnosed with depression who were referred for a polysomnogram (PSG) during a one-year period. Patients were diagnosed with obstructive sleep apnea (OSA) if their apnea-hypopnea index (AHI) was ≥5. Using the PSG monitoring results, patients were classified into two distinct groups: the OSA group, consisting of 50 patients, and the non-OSA group, which included 109 patients. An in-depth analysis was subsequently conducted on the sleep architecture and factors associated with the risk of OSA.

**Results:**

Among the 159 depression patients who met the subject criteria, 31.4% were diagnosed with OSA. Statistically significant differences were observed between the OSA group and the non-OSA group in terms of sex, body mass index (BMI), smoking status, and lipid levels (all p<0.05). The PSG monitoring results indicated that both the duration of non-rapid eye movement stage 3 (N3) sleep and the percentage of N3 sleep relative to total sleep time (N3/TST) were markedly lower in the OSA group than in the non-OSA group, with a statistically significant difference (p<0.01). Statistically significant differences were observed between the two groups regarding the number of awakenings, arousal index (ArI), mean oxygen saturation, oxygen saturation nadir, and oxygen desaturation index (all p<0.01). Multiple logistic regression analysis indicated that advanced age (odds ratio [OR]=1.034, 95% confidence interval [CI]: 1.004-1.065, p=0.024), female sex (OR=0.378, 95% CI: 0.171-0.837, p=0.016), low-density lipoprotein cholesterol (LDL-C) (OR=1.946, 95% CI: 1.026-3.692, p=0.041), and BMI≥25.0 kg/m² (OR=3.434, 95% CI: 1.411-8.360, p=0.007) were factors associated with OSA among patients with depression.

**Conclusion:**

OSA was prevalent among patients with depression. Risk factors for OSA included male sex, advancing age, a BMI≥25.0 kg/m², and elevated LDL-C levels.

## Introduction

Obstructive sleep apnea (OSA) is a complex disorder characterized by the recurrent collapse of the upper airway and airflow cessation during sleep (1). OSA profoundly impacts quality of life. Oxygen desaturation, hypercapnia, and sleep fragmentation linked to OSA trigger various cardiovascular and metabolic problems. This condition significantly increases the risk of hypertension, coronary artery disease, arrhythmias, heart failure, and stroke. Moreover, excessive daytime sleepiness and cognitive impairment resulting from OSA increase the likelihood of traffic or industrial accidents and create occupational challenges (2). The clinical symptoms of OSA include witnessed apneas, snoring, choking or gasping episodes, excessive daytime sleepiness, nonrestorative sleep, nocturia, sleep fragmentation, sleep maintenance insomnia, altered total sleep duration, morning headaches, loss of libido, irritability, and reduced concentration and memory (3). While these clinical symptoms might indicate a potential OSA diagnosis, none of them are pathognomonic of the disorder. Moreover, some of these symptoms are also observable in individuals with mental pathologies, such as depression.

Depression is a common psychiatric disorder worldwide and it has a broad influence on individual and societal health (4). It is estimated that approximately one in six individuals will experience the onset of depression at some point in their lives (5). Given the rising prevalence of depression, the prevention and management of its associated complications or comorbidities, such as obstructive sleep apnea (OSA), have become matters of utmost importance. Both conditions can cause fatigue and a sense of low energy, making daily activities arduous. The overlap of symptoms between depression and OSA can complicate the diagnosis and treatment process and may even result in OSA being underdiagnosis in patients with mental health issues. Thus, the association between OSA and depression deserves careful examination. Previously reported studies have indicated that the relationship between OSA and depression is bidirectional (6). OSA causes frequent arousal coupled with poor sleep quality, which may cause sleep fragmentation and chronic intermittent hypoxia resulting in the development or exacerbation of depression. Primavera D et al. reported OSA patients experience significantly more frequent depressive episodes, which in turn markedly impair their quality of life (7). On the other hand, the neurotransmitter dysregulation, disrupted circadian rhythm, and oxidative stress noted in patients with depression are likely to render them more susceptible to OSA (8).

Reported risk factors for obstructive sleep apnea (OSA) include sex, age, obesity, snoring and/or witnessed apnea during sleep, pharyngeal abnormalities, and cephalometric features (9). Shared risk factors such as obesity, a sedentary lifestyle, and aging may contribute to the co-occurrence of OSA and depression (10). The majority of these studies have employed questionnaires, actigraphy, or portable monitoring rather than the gold-standard method of attended polysomnography (PSG). Notably, most of these investigations have been conducted in Western countries, where obesity is highly prevalent. This factor could substantially influence the prevalence of OSA among patients with depression. In China, there is less research on the prevalence of OSA among patients with depression. Therefore, our study intends to evaluate the prevalence of OSA and its associated risk factors within this patient group.

## Method

### Participants

This study conducted a retrospective evaluation of the medical records of inpatients diagnosed with depression at the Sleep Department of The Affiliated Kangning Hospital of Ningbo University from January 2023 to December 2023. Patients were included in this study if they met the following criteria: 1) aged 18 years or older, 2) had a depressive episode meeting the diagnostic criteria of the International Classification of Diseases, Tenth Edition (ICD-10) and 3) completed sleep laboratory monitoring. The exclusion criteria were the presence of mental disorders other than depression, such as current or past mania; generalized anxiety disorder; obsessive–compulsive disorder; the presence of uncontrolled heavy physical disease; the presence of chronic pulmonary diseases; the presence or history of cranial trauma; the presence or history of central nervous system injury that could involve respiratory centers in the brain; the presence of predominantly central apnea syndrome; the presence of narcolepsy or primary hypersomnia; and the presence of a history of drug abuse.

The study received approval from the Ethics Committee of The Affiliated Kangning Hospital of Ningbo University (Approval NO.: NBKNYY- 2024-LC-46). Given that this was a retrospective, noninterventional study with all the data collected anonymously, informed consent was not necessary. The ethics committee waived the requirement of written informed consent for participation.

At the time of the study, demographic data, including age, sex, occupation, marital status, smoking behavior, and alcohol consumption, were collected. Clinical parameters, including height, weight, and lipid profiles (such as triglycerides, low-density lipoprotein cholesterol (LDL-C), high-density lipoprotein cholesterol (HDL-C), and total cholesterol), were likewise evaluated. Additionally, body mass index (BMI) was calculated via the formula BMI=weight (kg)/(height (m)*height (m)), which is the weight in kilograms divided by the square of the height in meters.

### Polysomnography data acquisition and analysis

In this study, overnight sleep monitoring was performed using the Somnoscreen™Plus V5 (Somnomedics, Germany) polysomnography system. PSG recordings included the following physiological signals: electroencephalogram (EEG, electrodes placed according to the 10-20 system), electrooculogram (EOG), electromyogram (EMG), electrocardiogram (ECG), nasal airflow, thoracoabdominal respiratory movements, oxygen saturation (SpO_2_), and body position. All signals were recorded synchronously at a sampling rate of 200 Hz, with the option to increase to 1000 Hz for high-resolution studies. Sleep monitoring was conducted in a standardized sleep laboratory environment. Participants were monitored from 22:00 to 07:00 the following morning. During the monitoring period, the laboratory was kept quiet, with controlled lighting and minimal external disturbances. Data acquisition was performed by trained technicians, and signal calibration and quality control were strictly conducted in accordance with the American Academy of Sleep Medicine (AASM) guidelines. PSG data were gathered, comprising total sleep time (TST), sleep efficiency (SE), wake after sleep onset (WASO), sleep latency (SL), rapid eye movement sleep (REM), sleep latency of REM (SLrem), nonrapid eye movement stage 1 (N1), nonrapid eye movement stage 2 (N2), nonrapid eye movement stage 3 (N3), mean oxygen saturation, oxygen saturation nadir, oxygen desaturation index, arousal index (ArI), and periodic leg movements during sleep (PLMS) data. The participants were categorized into two groups according to the presence or absence of obstructive sleep apnea (OSA). All respiratory events were scored using the American Academy of Sleep Medicine (AASM) criteria for polysomnography. Events were classified as hypopneas (HPs) when a decrease of 50% or more in nasal/oral airflow (N/O) and the chest signal was detected. In this work, the criterion for OSA based on the apnea–hypopnea index (AHI) followed the International Classification of Sleep Disorders, 3rd edition (ICSD-3), which requires an AHI of at least 5 events per hour as a cutoff for OSA. Moreover, an oxygen desaturation index of 4% (ODI of 4%) was regarded as significant if it occurred at least 5 times per hour, as measured by a pulse oximeter (2).

### Questionnaires

Upon admission, all patients underwent assessment of the severity of their subjective complaints of depression and anxiety, with the use of examiner-rating scales. Depressive symptoms were evaluated via the Hamilton Rating Scale for Depression (HAMD). This scale consists of 24 items (10 items are defined from 0 to 2, and 14 items are defined from 0 to 4). The final score can vary from 0 to 76. Questions of 0-2 points were defined as none (0), mild-moderate (1), or severe (2). Questions of 0-4 points were defined as none (0), mild (1), moderate (2), severe (3), and very severe (4). A final score of 0-7 indicates an absence of depression, 8-19 indicates mild depression, 20-34 indicates moderate depression, and a score ≥35 indicates severe depression (11). The presence of anxiety symptoms was explored through the use of the Hamilton Rating Scale for Anxiety (HAMA). Each item on this scale is scored on a numeric scale ranging from 0 (not present) to 4 (severe). The resulting final score can range anywhere from 0-56. A score above 17 is regarded as an indication of mild anxiety, whereas a score within the range of 25-30 is deemed to represent moderate-severe anxiety (12).

### Statistical analysis

All primary statistical comparisons were performed using IBM SPSS Statistics 26 (IBM Corp., Armonk, NY, USA), while effect sizes and 95% confidence intervals were calculated using Python 3.9 (Python Software Foundation). In this study, the proportion of missing data included in the analysis was relatively low (up to 5.7%), involving total cholesterol, HAMA and HAMD. Due to the skewed distribution of the data, the median imputation method was applied. Furthermore, other scales, such as PSQI and SCL-90, had a higher proportion of missing values and were therefore excluded from the analysis. For each categorical variable, the number and percentage (n, %) were used for description. Continuous variables were characterized by the mean and standard deviation (SD) if they followed a normal distribution, or by the median and interquartile range (IQR; 25th-75th percentiles) if they were skewed. The normality of continuous variables was assessed using the Shapiro-Wilk test. For continuous variables, the independent samples t-test was used to compare groups if the data followed a normal distribution, while the Wilcoxon-Mann-Whitney U test (U test) was employed for nonnormally distributed data. To compare categorical variables between two groups, the chi-square test for independence was utilized. Multiple logistic regression was performed to identify which characteristics were jointly associated with the diagnosis of OSA. To control for potential confounders, we performed Forward Stepwise Logistic Regression (FSLR). This approach began with the initial inclusion of all potential confounders, such as age, gender, BMI, and clinical covariates. Variables were then iteratively added or removed based on their statistical significance, with a threshold of p<0.05 for entry and p>0.10 for removal. Finally, the model was adjusted for significant confounders to ensure robust and reliable results. For all the statistical tests, a significance level of 5% (p=0.05) was set.

## Results

### Demographic characteristics and clinical parameters (**Table 1**)

**Table 1:** Univariate analysis of demographic and clinical parameters in the OSA group (50 patients) and the non-OSA group (109 patients).

	Non-OSA (*n* = 109)	OSA (*n* = 50)	Test Method	Effect Size	95% CI		Statistical coefficients	P-value
					Lower Limit	Upper Limit		
Gender, n (%)								
Male	36 (56.3%)	28 (43.8%)	Chi-square	Phi = 0.217	0.123	0.311	χ² = 7.522	0.006
Female	73 (76.8%)	22 (23.2%)						
BMI (kg/m²) (*x*+*s*)	21.8+3.2	24.3+3.9	t-test	Cohen's d =-0.681	-3.649	-1.339	t = -4.266	<0.001
Age (years) M (P25, P75)	43.0 (32.3, 55.0)	51.0 (37.0, 58.0)	U test	r = -0.120	-8.432	1.057	z = -1.520	0.129
Occupation, n (%)								
Unemployed	54 (75.0%)	18 (25.0%)	Chi-square	Phi = 0.126	0.023	0.229	χ² = 2.537	0.111
Employed	55 (63.2%)	32 (36.8%)						
Marital status, n (%)								
Single	33 (75.0%)	11 (25.0%)	Chi-square	Phi = 0.126	0.028	0.224	χ² = 1.173	0.279
Married	76 (66.1%)	39 (33.9%)						
Cigarette use, n (%)								
None	104 (71.2%)	42 (28.8%)	Chi-square	Phi = 0.169	0.045	0.293	χ² = 4.524	0.033
Yes	5 (38.5%)	8 (61.5%)						
Alcohol use, n (%)								
None	105 (70.0%)	45 (30.0%)	Chi-square	Phi = 0.127	-0.024	0.276	χ² = 1.523	0.217
Yes	4 (44.4%)	5 (55.6%)						
TG (mmol/L), M (P25, P75)	1.1 (0.8, 1.6)	1.2 (1.0, 2.0)	U test	r = -0.169	-3.989	0.864	z =- 2.129	0.033
TC (mmol/L), M (P25, P75)	4.4 (4.0, 5.0)	4.7 (4.1, 5.3)	U test	r = -0.148	-0.538	0.033	z = -1.868	0.062
LDL-C(mmol/L), M (P25, P75)	2.4 (2.1, 2.9)	2.7 (2.3, 3.1)	U test	r = -0.195	-0.467	-0.043	z = -2.465	0.014
HDL-C(mmol/L), M (P25, P75)	1.3 (1.1, 1.4)	1.2 (1.0, 1.3)	U test	r = 0.171	-0.009	0.178	z = -2.156	0.031
HAMA score, M (P25, P75)	26.0 (19.0, 30.0)	27.0 (19.0, 33.0)	U test	r = -0.051	-3.635	1.756	z = -0.650	0.516
HAMD score, M (P25, P75)	30.0 (24.0, 36.8)	31.0 (25.0, 38.5)	U test	r = -0.018	-4.004	2.623	z = -0.232	0.816

The current study included 159 patients suffering from depression. The median age of the participants was 46 (34, 56) years, and 40.3% of them were male. In terms of the PSG results, the median AHI for the entire sample was 3.0 (1.7, 6.7) events per hour. Notably, the median AHI of the OSA group was significantly greater, reaching 11.9 events per hour, as opposed to 2.2 events per hour among patients without OSA. The median age of the OSA group was 43 (32.5, 55.5) years, whereas that of the non-OSA group was 50 (37.5, 57.3) years. However, there was no significant difference in age between the two groups. Among the patients with depression in our sample, 50 had OSA. This group consisted of 28 males and 22 females, with a prevalence of 31.4%. When analyzed by sex, OSA was diagnosed in 43.8% of the men (28 out of 64) and 23.2% of the women (22 out of 95). A chi-square analysis of categorical variables indicated a significant sex difference. Specifically, a greater proportion of men than women had an AHI≥5. The proportion of smokers diagnosed with OSA was 61.5%, which was significantly greater than that of nonsmokers (28.8%). In contrast, no significant difference was detected in the OSA diagnosis rates between drinkers (55.6%) and nondrinkers (30.0%). Following the assessment of age, sex, smoking, and alcohol consumption in the OSA group and the non-OSA group, we compared other demographic variables between the two groups and found no significant differences in terms of marital status or occupation.

In terms of anthropometric measurements, a statistically significant difference in BMI was observed between the OSA group and the non-OSA group. The mean BMI of the OSA group was 21.8 kg/m², whereas that of the non-OSA group was 24.3 kg/m². Regarding the threshold for overweight (BMI≥25.0 kg/m²), OSA was diagnosed in 55.6% (20 out of 36) of patients with a BMI≥25.0 kg/m², as opposed to only 24.4% (30 out of 123) of patients with a BMI<25.0 kg/m².

Among the blood parameters, while the difference in total cholesterol did not reach statistical significance (p=0.062, approaching significance), the lipid profile demonstrated notable differences between the OSA group and the non-OSA group. In patients with OSA, triglycerides and LDL-C were significantly elevated, whereas HDL-C levels were lower than those in patients without OSA.

For the rater-rated scales, namely, the HAMD and HAMA, no significant differences were found between the OSA group and the non-OSA group in our survey.

### Polysomnography monitoring results (**Table 2**)

**Table 2:** Univariate analysis of polysomnography (PSG) monitoring results between the obstructive sleep apnea group (50 patients) and the non-obstructive sleep apnea group (109 patients).

	Non-OSA (*n* = 109)	OSA (*n* = 50)	Effect Size	95% CI		Statistical coefficients	P*-*value
				Lower Limit	Upper Limit		
TST (min), M (P25, P75)	477.0 (421.0, 531.2)	495.5 (440.5, 534.5)	r = -0.230	-0.438	-0.032	z = -0.755	0.439
SE (min), M (P25, P75)	84.7 (76.0, 91.3)	82.6 (76.1, 88.0)	r = -0.127	-0.314	0.060	z = -1.586	0.113
WASO (min), M (P25, P75)	56.7 (32.4, 100.0)	73.5 (46.2, 110.2)	r = -0.129	-0.313	0.055	z = -1.643	0.100
the number of awakenings, M (P25, P75)	26.0 (16.0, 37.5)	33 (21.5, 46.8)	r = -0.206	-0.389	-0.023	z = -2.603	0.009
SL (min), M (P25, P75)	18.0 (9.0, 31.5)	18.5 (7.3, 36.3)	r = -0.161	-0.341	0.019	z = -0.043	0.966
SLrem (min), M (P25, P75)	203.0 (142.8, 293.3)	197.3 (111.5, 304.3)	r = -0.024	-0.201	0.153	z = -0.302	0.762
N1(min), M (P25, P75)	44.7 (31.3, 62.2)	51.0 (40.6, 70.2)	r = -0.150	-0.327	0.027	z = -1.884	0.059
N2(min), M (P25, P75)	344.7 (286.3, 400.7)	339.1 (305.8, 383.7)	r = -0.084	-0.261	0.093	z = -0.052	0.959
N3(min), M (P25, P75)	13.2 (5.9, 43.3)	5.3 (0, 30.7)	r = -0.230	-0.438	-0.032	z = -2.893	0.004
REM(min), M (P25, P75)	54.9 (36.3, 81.6)	57.0 (41.3, 82.3)	r = -0.056	-0.232	0.120	z = -0.692	0.489
N1/TST (%), M (P25, P75)	8.8 (6.7, 13.6)	10.6 (8.3, 15.1)	r = -0.135	-0.315	0.045	z = -1.673	0.094
N2/TST (%), M (P25, P75)	73.1 (64.9, 79.4)	71.5 (64.7, 77.8)	r = -0.047	-0.223	0.129	z = -0.595	0.552
N3/TST (%), M (P25, P75)	2.9 (1.35, 8.35)	1 (0, 6.1)	r = -0.230	-0.438	-0.032	z = -2.913	0.004
REM/TST (%), M (P25, P75)	11.4 (7.6, 16.4)	12.1 (7.7, 18.7)	r = -0.049	-0.225	0.127	z = -0.616	0.538
mean oxygen saturation, M (P25, P75)	96.0 (95.0, 97.0)	95.0 (93.0, 96.0)	r = -0.360	-0.556	-0.164	z = -4.699	<0.001
oxygen saturation nadir, M (P25, P75)	91.0 (89.0, 93.0)	86.0 (82.8, 88.0)	r = -0.590	-0.778	-0.402	z = -7.457	<0.001
oxygen desaturation index, M (P25, P75)	2.9 (1.5, 5.8)	16 (9.5, 31.3)	r = -0.628	-1.152	-0.104	z = -7.910	<0.001
ArI, M (P25, P75)	15.4 (10.2, 23.1)	19.6 (13.8, 33.0)	r = 7.065	6.727	7.403	z = -3.044	0.002
PLMS, M (P25, P75)	0.7 (0, 4.0)	1.4 (0, 4.1)	r = 9.808	9.471	10.146	z = -0.311	0.756

PSG monitoring revealed that, compared with patients without OSA, patients in the OSA group had a significantly longer duration of N3 sleep (p=0.004) and a significantly higher proportion of N3 sleep within the sleep cycle (N3/TST and N3/SPT; p=0.004), whereas the number of awakenings also significantly increased (p=0.009). With respect to various sleep parameters, including total sleep time (TST), sleep period time (SPT), sleep latency (SL), rapid-eye-movement (REM) sleep latency (SLrem), sleep efficiency (SE), N2 sleep, rapid-eye-movement (R) sleep, the ratios of N1 sleep to TST (N1/TST) and SPT (N1/SPT), the ratios of N2 sleep to TST (N2/TST) and SPT (N2/SPT), the ratios of R sleep to TST (R/TST) and SPT (R/SPT), wake after sleep onset (WASO), and periodic limb movements in sleep (PLMS), there were no significant differences between the OSA group and the non-OSA group. However, this was not the case for the arousal index (ArI). The median ArI was 19.6 (13.8, 33.0) greater in the OSA group than in the non-OSA group, with a median of 15.4 (10.2, 23.1) (p=0.002). Additional data from the PSG revealed significant differences between the two groups in terms of the mean oxygen saturation, oxygen saturation nadir, and oxygen desaturation index (a 4% or greater decrease in oxygen saturation per hour), with p values<0.001. Moreover, the OSA group presented higher levels of N1 and WASO than the non-OSA group did, but these differences did not reach statistical significance (p=0.094 and 0.100, respectively).

### Multiple logistic regression (**Table 3**)

**Table 3:** Forward stepwise logistic regression analysis of risk factors associated with obstructive sleep apnea in patients with depression.

Variable	Group	Coefficient (B)	Standard Error (S.E.)	Walds χ²	p-value	Odds Ratio (OR)	95%Cl	
							Lower	Upper
Age		0.033	0.015	5.094	0.024	1.034	1.004	1.065
Gender	Male*							
	Femal	-0.973	0.406	5.753	0.016	0.378	0.171	0.837
LDL-C		0.666	0.327	4.159	0.041	1.946	1.026	3.692
BMI	<25.0*							
	≥25.0	1.234	0.454	7.390	0.007	3.434	1.411	8.360
Constant		-3.879	1.148	11.431	0.001	0.021		

*Control groups.

Forward stepwise logistic regression (FSLR) was employed to evaluate which characteristics were jointly associated with the diagnosis of OSA. The Hosmer-Lemeshow goodness-of-fit statistic (χ²=5.67, df=8, p=0.684) indicated the adequacy of the forward stepwise (likelihood ratio) binary logistic regression model. In our study, when males were set as the reference group, the OR=0.378 indicates that females have 0.378 times the risk of developing OSA compared to males. This means that females have a significantly lower risk of OSA than males, with a risk reduction of approximately 62.2%. Logistic regression analysis revealed that age, male sex, LDL-C, and BMI (considered dichotomous variables with a cutoff of 25.0) were independent predictors of the risk of OSA in patients with depression (p<0.05). Notably, high-risk patients were significantly older, more frequently male, a greater proportion of them had a BMI≥25.0 kg/m2, and their LDL-C levels were higher.

## Discussion

Many prior studies have focused on the prevalence of depression among patients with obstructive sleep apnea (OSA) (13–15). In contrast, this study specifically investigated the prevalence of OSA, as well as the demographic and clinical characteristics associated with its risk, in patients with depression. Research has reported that the incidence of OSA in patients with depression is approximately 11%-18%, which is higher than that reported in the general population (16). According to a particular study of adult patients with depression carried out in 2020, the prevalence of OSA, defined by an AHI≥5/h according to the AASM criteria, reached 36.3% within the patient population (17). Ong JC et al. reported that the prevalence of OSA was 39% among a group of 51 individuals with major depression (18). In comparison, the prevalence among community-screened patients was merely 2-14% (19). Another study reported that, among 703 patients with depression, the prevalence of moderate to severe OSA (defined by an AHI≥15/h) was 13.9% (20). A bidirectional Mendelian randomization study provided evidence that genetically predicted depression was significantly associated with an increased risk of OSA (10). In our sample of individuals with depression, the prevalence of OSA was 31.4%. This prevalence is comparable to that reported in the study by Stubbs B et al. (17), yet it is significantly higher than that reported in the general population. One possible reason for this high prevalence is that patients with depression tend to be prone to obesity. This is due to factors such as reduced outdoor exercise, diminished self-care ability, and the use of medications such as antidepressants. In fact, individuals grappling with depression or anxiety are significantly more predisposed to adopting unfavorable lifestyle changes compared to the general population, such as smoking, alcohol consumption, and less outdoor exercise (9). Some specific antidepressants are closely associated with a heightened risk of weight gain. Notably, weight gain is a well-established risk factor for the development of OSA. Varadharajan N et al. reported that One-fifth of the patients with BD was found to be at high risk for OSA, compared to the 6% prevalence in the control group (21). Studies have suggested that the neurotransmitter dysfunction, altered circadian rhythm, and oxidative stress observed in patients with depression might confer increased susceptibility to OSA (22). Depression is frequently associated with decreased levels of 5-hydroxytryptamine (5-HT). This is considered one of the key mechanisms leading to upper respiratory collapse and may ultimately give rise to OSA (23). Many studies on OSA patients have revealed a positive relationship between the AHI and depression symptom severity (24–26). However, we did not find a relationship between HAMD scores and OSA, which is consistent with reports in the literature indicating that the severity of depression is not a risk factor for OSA (17). Prior studies (27) have identified OSA not only as a significant predictor of serious adverse outcomes following stroke or ischemic heart disease (IHD) but also as closely associated with metabolic syndrome (MetS), where it has been reported that OSA increases the risk of MetS by 72% (28). Additionally, OSA is not only associated with depressive symptoms but also may cause a lack of response to antidepressant pharmacotherapy (29, 30). Therefore, for individuals with depression, identifying the specific risk factors for OSA is crucial. This can enhance the detection and management of this syndrome and reduce complications for these patients.

The classical risk factors for OSA in the general population include age, male sex, obesity, snoring, high blood pressure, metabolic syndrome, and a sleep duration of 8 hours or more (31–34). Among these factors, an increase in BMI is associated with a greater incidence of OSA (35), although obesity has been reported to be less useful as a risk factor for OSA in the Asian population (36). Similarly, in our study, we identified male sex, age, and BMI as risk factors for OSA in the subpopulation of patients with depression. We also found that the prevalence of OSA in men was significantly greater than that in women. Women reported less snoring and fewer episodes of respiratory events than men did, whereas insomnia, fatigue, headaches, and mood changes were reported more frequently among women. This discrepancy may lead to the underdiagnosis of OSA in females (37). In adults, the frequency of sleep-disordered breathing increases with age (38). In our study, while nonparametric tests revealed no significant difference in age between the two groups-likely due to the influence of confounding factors-logistic regression analysis revealed that age was a risk factor for OSA in patients with depression. Obesity, as measured by body mass index (BMI), is one of the primary risk factors for OSA in patients (39). Our study demonstrated that an increase in BMI elevates the risk of developing OSA. The mean BMI of the study cohort was 22.3 kg/m², and there was a significant difference between patients with OSA (24.3 kg/m²) and those without OSA (21.8 kg/m²). In our study, we found that being overweight (BMI≥25.0 kg/m²) is an independent risk factor for OSA in depressed patients. When BMI is treated as a continuous variable in regression analysis, fewer risk factors are identified. However, converting BMI into a binary variable (using a cutoff of 25.0) reveals more risk factors. In summary, the discovery of more risk factors when BMI is dichotomized suggests the presence of nonlinear or threshold effects in our data, or that a binary variable is better suited for the current analysis. This finding highlights the importance of considering alternative modeling approaches, such as dichotomization, to better capture the relationship between BMI and the outcome of interest in our study. Tündüz C et al. reported that elevated triglyceride (TG), total cholesterol (TC), and low-density lipoprotein cholesterol (LDL-C) levels were commonly found in patients with OSA, whereas high-density lipoprotein cholesterol (HDL-C) levels were decreased (40). Our results were similar. However, the difference in TC between the two groups did not reach statistical significance (p=0.062). Dyslipidemia is closely linked to obesity, which is acknowledged as one of the primary risk factors for OSA (41). Conversely, other studies have identified a positive association between OSA and lipid homeostasis, independent of obesity (42, 43). This study reported that LDL-C is an independent risk factor for OSA(OR=1.946, 95 % CI=1.026-3.692) in patients with depression, a finding that aligns with previous research in the general population, where LDL-C has also been identified as an independent risk factor for OSA (OR=1.430, 95% CI=1.221-1.675) (44). However, due to shared pathophysiological mechanisms such as chronic inflammation and hypothalamic-pituitary-adrenal (HPA) axis dysregulation, the magnitude of effect (e.g., odds ratios) of these risk factors may be amplified in patients with depressive disorders compared to the general population. This suggests that while the associations between risk factors and obstructive sleep apnea (OSA) are consistent between the general population and patients with depressive disorders, the biological vulnerability inherent in depressive disorders may potentiate their clinical impact.

Alcohol, which acts as a central nervous system inhibitor and causes peripheral muscle relaxation, theoretically plays a crucial role in regulating the incidence and severity of OSA. The guidelines of the American Academy of Sleep Medicine (AASM) advise against the use of alcohol before bedtime in patients with OSA (3). Alcohol consumption deteriorates sleep-related breathing parameters, such as the apnea-hypopnea index (AHI) and mean oxyhemoglobin saturation (SpO_2_), particularly in individuals with OSA and a history of snoring (45). Smoking impacts OSA through diverse mechanisms. These include alterations in sleep structure, upper respiratory tract neuromuscular function, arousal mechanisms, and an increase in upper respiratory tract inflammation (46). A study involving 3,791 participants revealed that even though an independent effect of smoking on OSA was not found, the daily cigarette count, pack-years, and scores of the Fagerstrom Test for Nicotine Dependence were higher in patients with more severe OSA (47). In this study, although a statistically significant difference in smoking was observed between the OSA group and the non-OSA group, we determined that neither alcohol nor smoking was a risk factor for OSA in the subpopulation of individuals with depression. This might be attributed to the fact that our study was a cross-sectional study, and some patients ceased drinking and smoking after developing depression.

When our results are interpreted, several limitations should be taken into account. First, a major limitation of this study lies in its retrospective design. This design inherently poses challenges in establishing cause-and-effect relationships. Retrospective research depends on historical data, which are vulnerable to various biases and other confounding factors. Although we made efforts to address these potential issues, the resulting findings merely demonstrate associations rather than causal links. To gain a more in - depth understanding of these relationships, future investigations should adopt prospective or experimental designs. Second, rather than being a cross-sectional community sample of participants diagnosed with depression, the study cohort was selected from the sleep department of a psychiatric hospital. This selection method may introduce center-specific biases, thereby undermining the generalizability of our findings. Furthermore, it is plausible that certain patients who would otherwise have satisfied the criteria for inclusion in the study cohort were precluded from participation because of the loss or incompleteness of data within the medical records. Third, in this study, owing to the employment of single-night polysomnography (PSG) monitoring, sleep architecture parameters are likely to be influenced by the "first-night effect". Manifestations may include prolonged sleep latency, decreased sleep efficiency, and disrupted sleep architecture, all of which have the potential to undermine the accuracy of the results. Despite this, a study by Ma J et al. demonstrated that among adult Chinese snorers undergoing two consecutive nights of PSG monitoring, only a mild first-night effect (FNE) was observed, which did not affect the diagnosis of obstructive sleep apnea hypopnea syndrome (OSAHS) (48). Future studies could use multiple-night assessments to minimize this effect and obtain more reliable data. Fourth, medications such as antidepressants and benzodiazepines (BZDs) were not included in our study. These drugs have the potential to exacerbate obstructive sleep apnea. Specifically, BZDs can trigger upper-airway collapse, which may in turn affect respiration. For instance, use of clonazepam has been reported to induce OSA (49). Finally, although patients suffering from hypoxic diseases such as cardiopulmonary disorders were excluded, the absence of objective clinical examinations represents a notable limitation. Despite these limitations, a pivotal strength of this research lies in the use of laboratory-based PSG monitoring to assess all obstructive OSA indices instead of relying on proxy measures such as witnessed apnea or portable PSG. In future research, increasing the sample size could render the results more precise and compelling.

## Conclusion

Our findings demonstrate that obstructive sleep apnea (OSA) is highly prevalent among patients afflicted with depression. Additionally, male gender, increasing age, BMI≥25.0 kg/m², and elevated LDL-C levels were identified as significant risk factors for the diagnosis of OSA in these patients.

## Data Availability

The raw data supporting the conclusions of this article will be made available by the authors, without undue reservation.
